# *Children*, parents, and pets exercising together (CPET) randomised controlled trial: study rationale, design, and methods

**DOI:** 10.1186/1471-2458-12-208

**Published:** 2012-03-19

**Authors:** Philippa S Yam, Ryan Morrison, Viki Penpraze, Carri Westgarth, Dianne S Ward, Nanette Mutrie, Pippa Hutchison, David Young, John J Reilly

**Affiliations:** 1 University of Glasgow College of Medical, Veterinary, and Life Sciences, Glasgow, UK; 2 University of Liverpool Department of Epidemiology and Population Health, Institute of Infection and Global Health, Leahurst Campus, Neston CH64 7TE, UK; 3 University of North Carolina at Chapel Hill, Department of Nutrition, Gillings School of Global Public Health, Chapel Hill 27599-7461, NC, USA; 4 University of Strathclyde Physical Activity for Health Group, School of Psychological Sciences and Health, Glasgow G13 1PP, UK; 5 University of Strathclyde Dept. of Mathematics and Statistics, Livingstone Tower, Glasgow, UK

**Keywords:** Obesity, children, dogs, accelerometry, exercise, CPET

## Abstract

**Background:**

Objectively measured physical activity is low in British children, and declines as childhood progresses. Observational studies suggest that dog-walking might be a useful approach to physical activity promotion in children and adults, but there are no published public health interventions based on dog-walking with children. The Children, Parents, and Pets Exercising Together Study aims to develop and evaluate a theory driven, generalisable, family-based, dog walking intervention for 9-11 year olds.

**Methods/design:**

The Children, Parents, and Pets Exercising Together Study is an exploratory, assessor-blinded, randomised controlled trial as defined in the UK MRC Framework on the development and evaluation of complex interventions in public health. The trial will follow CONSORT guidance. Approximately 40 dog-owning families will be allocated randomly in a ratio of 1.5:1 to receive a simple behavioural intervention lasting for 10 weeks or to a 'waiting list' control group. The primary outcome is change in objectively measured child physical activity using Actigraph accelerometry. Secondary outcomes in the child, included in part to shape a future more definitive randomised controlled trial, are: total time spent sedentary and patterning of sedentary behaviour (Actigraph accelerometry); body composition and bone health from dual energy x-ray absorptiometry; body weight, height and BMI; and finally, health-related quality of life using the PedsQL. Secondary outcomes in parents and dogs are: changes in body weight; changes in Actigraph accelerometry measured physical activity and sedentary behaviour. Process evaluation will consist of assessment of simultaneous child, parent, and dog accelerometry data and brief interviews with participating families.

**Discussion:**

The Children, Parents, and Pets Exercising Together trial should be the first randomised controlled study to establish and evaluate an intervention aimed at dog-based physical activity promotion in families. It should advance our understanding of whether and how to use pet dogs to promote physical activity and/or to reduce sedentary behaviour in children and adults. The trial is intended to lead to a subsequent more definitive randomised controlled trial, and the work should inform future dog-based public health interventions such as secondary prevention interventions in children or adults.

**Trial registration number:**

ISRCTN85939423

## Background

Recent studies which have measured physical activity objectively show that few primary school-age children in the UK meet public health recommendations for physical activity of one hour of moderate-vigorous intensity physical activity (MVPA) daily [1-4]. Low levels of physical activity can be expected to have deleterious effects with substantial public health impact [5]. Physical activity is even lower than UK averages for children for some ethnic minority groups [6], lower in girls than boys, and lower among the overweight and obese [4]. Recent UK studies have shown that objectively measured physical activity declines, and objectively measured sedentary behaviour increases before adolescence [4,7]: these changes in mid-late childhood are also more marked among girls, the overweight and obese, those with lowest levels of physical activity [4], and predict subsequent changes in body fatness [8]. Moreover, recent evidence has shown that of all the periods from birth to late adolescence, mid-late childhood in the UK (around the ages of 7-11 years) is characterised by the greatest incidence (emergence of new cases) of obesity [9], and the greatest degree of excessive weight gain among those who do not become obese [10]. This recent evidence suggests that mid-late childhood (towards the end of the primary school years in the UK) should be considered a priority in interventions aimed at promoting physical activity, preventing future obesity, and/or promoting a reduction in sedentary behaviour.

Systematic reviews of interventions to promote physical activity in school-age children suggest that physical activity is modifiable to some degree [11,12], and physical activity promotion is helpful to obesity prevention [13,14]. Physical activity is potentially very flexible in children, influenced largely by factors in the physical and cultural environment which are at least partly modifiable [15]. Making environmental changes which are conducive to physical activity is a logical response to concerns over the low levels of physical activity of children. However, systematic reviews on interventions to promote physical activity in childhood have noted many limitations in the evidence, and most interventions have had only modest and short-term impact on physical activity or obesity risk [11,12,15]. The belief that increased physical activity will reduce obesity risk has even been questioned [15-17]. The evidence on interventions to promote physical activity therefore suggests that novel approaches to physical activity promotion in childhood are required: these should aim to have more marked effects on physical activity, and should aim to be more sustainable than interventions which have been typical in the past.

Dog ownership is a significant societal factor that may be used to encourage and sustain health behaviour change at individual and population level [18]. Many UK households own dogs, and it is unlikely that they are all walked on a frequent and regular basis, thus providing a target group for interventions to promote physical activity. It has been suggested that the pet dog is a potentially valuable but neglected resource for physical activity promotion in children and adults [18], providing opportunities with wide population 'reach'. In Scotland for example there are approximately 800,000 dogs and 360,000 children of primary-school age. Approximately 20-30% of households in many western countries own pet dogs (19,). In the UK approximately 22-24% [19,20] of all households own a pet dog, but dog ownership is even more common among households with children [20]. A number of cross-sectional observational studies, including some studies which have measured physical activity objectively, have shown slightly higher physical activity levels for adults who walk their dogs regularly and have reported a tendency for adult dog owners to meet physical activity recommendations more commonly than non dog-owners [21-23]. Recent UK evidence suggests that, in the absence of any specific intervention, children typically are slightly more physically active if they own a dog [24]. However, one Australian study examined dog walking in children: only 23% of 5-6 year olds and 37% of 10-12 year old children ever walked with their dog [25]. Dog ownership has been associated with lower weight status and increased physical activity in some sub-groups within studies, but not all sub-groups [25-27]. A recent study across the city of Liverpool in the UK found that 59% of 9-10 y olds who owned dogs reported some involvement in walking their dogs, but only 34% reported walking their dogs daily (unpublished, Westgarth et al.). Recent analyses of the large UK birth cohort 'ALSPAC' (the Avon Longitudinal Study of Parents and Children) provided no evidence that 7 year old children whose family owned a dog were less likely to be overweight/obese [28]. One intervention study which used dogs to promote physical activity [29] was promising, but was an obesity treatment trial in adults only, and so is of limited relevance to public health interventions for children and their families.

In summary, dogs are present in a high proportion of households, and have the potential to increase child and adult physical activity [18], but have not been used for this purpose to any great extent to date. Promotion of more walking and play with the dog could be a useful strategy to promote family physical activity. At this stage however, evidence is lacking as to whether and how interventions with families and their dogs can be used to promote physical activity. There is sufficient evidence from observational studies now to conclude that dogs and their owners have potential to walk more, and intervention studies are now justified.

Concerns over declining physical activity in mid-late childhood suggest that this may be a useful period of the lifecycle to intervene to promote physical activity, or at least to mitigate the reduction in physical activity which occurs at this time. Most interventions to date have been school-based [11,12]. There is much less evidence from interventions in other settings, including the family/home [30,31], despite the evidence that the home environment is a consistent predictor of objectively measured physical activity level in studies of mid-late childhood [30-32], and the suggestion from a recent systematic review that the home/family may be the most promising setting for promoting child physical activity [11]. Approaches aimed at promoting physical activity should be developed and evaluated using a robust framework [33], should have a theoretical basis [33,34], and should be tested rigorously using randomised controlled trials (RCT; 33).

The current study, Children, Parents, and Pets Exercising Together (CPET) therefore aims to develop and evaluate a family-based physical activity promotion intervention based on using the pet dog to increase physical activity and reduce sedentary behaviour for the family. The intervention is theory-driven and the development of the study has followed advice in the UK Medical Research Council Framework on the Development and Evaluation of Complex Interventions [33], including the advice to carry out an 'exploratory trial' before carrying out a larger scale, longer-term, more 'definitive trial'. The present exploratory trial is therefore the first step along the path towards a definitive trial, leading to at least one more RCT, drawing on the UK Medical Research Council Framework and other guidance such as 'RE-AIM' [35]. The study should also inform the development of future public health interventions involving dogs in other populations (e.g. in adults, in older adults; 18). The specific research questions being asked in CPET are listed in Table 1.

**Table 1:** Research questions to be addressed in CPET study

	Research questions
**A: Trial recruitment and retention**	i) How feasible is the trial ii) Is recruitment realistic: are families willing to be allocated randomly iii) What is the extent of sample attrition iv) To what extent is the trial affected by missing data, perhaps arising from inconvenience of home visits or the necessity to make hospital visits for outcome measures including DXA scans

**B: Feasibility of the intervention and implications for future trials**	i) How feasible is the dog-based intervention ii) Is process evaluation favourable iii) How should intervention content and delivery be modified for future interventions in the light of study findings iv) What sample size would be required for a more definitive trial for each of the outcomes tested

**C: Efficacy**	i) Is there preliminary evidence of favourable outcomes for primary and/or secondary outcomes ii) Is there any indication that increases in physical activity during the intervention are compensated (it is not known whether increased physical activity in dog-walking would reduce physical activity at other times; 'compensation' (15,17) iii) Does physical activity undertaken as part of the dog walking intervention displace other forms of physical activity (e.g. going to the gym)

## Methods/design

### Study sample, recruitment, inclusion and exclusion criteria

It is intended to send invitation letters to participate in the study to children in primary school years 6 and 7 at all 36 primary schools in one local authority area, East Dunbartonshire, in the West of Scotland. East Dunbartonshire local authority provides a well defined geographical area for the aspects of the intervention which involve the wider environment, and this single local authority includes the entire spectrum of socio-economic status. Recruitment effort, participation rate, and retention rate will be documented.

Families will be included if they consent to participation, have children age 9-11 y, own a dog, if children and parents have no physical or intellectual impediment to participating, if at least one parent is willing to be the focus of the intervention (and be responsible for taking part in the intervention sessions and outcome measurement sessions), and if their dogs are deemed physically and psychologically safe to participate in the intervention.

### Ethical and safety considerations

The study has the approval of the University of Glasgow College of Medical, Veterinary, and Life Sciences Ethics Committee, which deals with ethical and safety aspects of the study from the perspective of the human participants and the researchers. Informed written consent to participation will be required from each participating child, and from each participating parent. The trial also has the approval of the University of Glasgow School of Veterinary Medicine Ethics and Welfare Committee, and the University of Liverpool Leahurst Research Ethics Committee, which consider the ethical, welfare, and safety aspects of the study on behalf of participating dogs and their owners.

Safety of the intervention will be enhanced by emphasising throughout the initial recruitment phase, and the intervention sessions, that the focus of the intervention is the family and the dog being active together- the intervention is not intended to promote children taking the dog outside the home without the parent. Judgement as to whether it is safe for the dog to be involved in the intervention will be based on a screening questionnaire developed by two full members of the Association of Pet Behaviour Counsellors (Westgarth and Hutchison, the latter also a Certified Clinical Animal Behaviourist), both involved in the study, and based on the professional assessment during the initial home visit intervention session. It is intended to exclude dogs which are physically unable to take part in the intervention (e.g. due to limitations in mobility), and to exclude dogs which are behaviourally unsuitable for the intervention because of their tendency towards nervousness, aggression, or other behaviours which might make the intervention unsafe (e.g. strong drive to chase).

### Design, randomisation, allocation concealment, blinding

This is an individual RCT, and will follow guidance on the conduct and reporting of RCT outlined in the CONSORT statement [36]. After baseline outcome measures are made, participating families will be allocated randomly to intervention or control group (in the ratio of 1.5: 1 respectively).

Allocation concealment will be ensured by separation of the process of allocation from the researchers involved in the outcome measures. Blinding of researchers who make the outcome measures will be achieved by having two researchers at separate sites, one responsible for carrying out the intervention and the other responsible for carrying out the outcome measures.

### Statistical analysis and sample size considerations

The proposal is for an exploratory study, intended in part to inform future sample size calculations for all of the outcome measures used. The effect of the intervention is unknown and there are no comparable intervention data to inform a power calculation. We aim to enter approximately 40 families: a study of this size would be appropriate for an exploratory trial and indeed would be larger than many previous physical activity intervention studies in childhood [11-13]. While no comparable data exist to inform a sample size calculation, if we use the primary outcome of change in child total volume of physical activity (accelerometry) and assume a moderate effect size, equivalent to the pooled mean paired difference between primary school age children with a variety of mild chronic diseases and matched healthy controls (using unpublished data from Penpraze et al., author) then a sample size calculation is possible. Our estimates involve an increase in daily accelerometry count in the intervention group equivalent to 187 counts/minute with no change in the control group, and an SD of 160 counts/minute for the difference in the change between groups. With 80% power at *p *= 0.05, this difference would be detectable in the present study with 15 families per group. The difference between groups is 18% when expressed as a difference in the change in accelerometry count per minute between intervention and control groups. This is not equivalent to a difference in total volume of physical activity of 18% as there is not a 1:1 relationship between total volume of physical activity and accelerometer count per minute [4]. The difference between groups in the change in physical activity assumed for the power calculation is equivalent to around a 6% difference (40 minutes/day) in total volume of physical activity between groups, equivalent to a difference in total time spent sedentary between groups of approximately 40 minutes per day [4]. This calculation suggests that the present study, though exploratory, would be powered to detect differences in the change in total volume of physical activity between groups which could be biologically meaningful.

Statistical analysis would be carried out on an intention to treat basis, with all families included in the groups to which they had been allocated originally. Missing data would be replaced using the last measure carried forward method.

There is one planned per-protocol analysis. Outcomes from families deemed by the researchers to be highly engaged (criteria being parent and child attendance in all three face-to-face intervention meetings) will be compared with those less engaged.

### Intervention and control groups

The intervention group will participate in a 10 week participant-informed and theory-based intervention which aims to increase the frequency, intensity, and duration of dog-walking/playing with the family dog via increased dog walking and physically active play (parent and child walking and playing with the dog together). We consider that all children who meet study inclusion criteria will be eligible, regardless of their baseline levels of physical activity. The rationale for this is that at present < 10% of UK children meet the recommendation of 60 minutes of daily MVPA [1-4] when physical activity is measured objectively, and so almost all participating children will be insufficiently physically active at baseline. Physical activity guidelines in the UK in 2011 also emphasised the benefits of accumulating > 60 minutes of MVPA daily and so even for children who meet the guideline at baseline additional physical activity would be beneficial.

The intervention will be individualised and participant-centred, using progressive individual targets agreed for dog walking/play with the dog for each family. Various levels of organisation (wider environment, but predominantly the family environment) will be used to promote physical activity of children, their parents, and their dogs. The intervention will rely most heavily on modification of the family environment, and the use of parental support for child physical activity in the light of the promise of this as an intervention strategy [11,30-32]-the main practical change sought is to bring about in the intervention is the family and dog walking/playing more outside the home (including the garden), and doing so together.

The intervention will use the principal client-centred behavioural change techniques which derive from social cognitive theory [30]. These include decisional balance, self-monitoring of dog walking/physically active play with the dog (including a simple dog walking activity chart), goal setting, rewards, behavioural contracting, problem solving, and relapse prevention. All of these techniques are well established in adult behaviour change interventions, and are also used widely in behaviour change interventions with children of this age [37]. Self monitoring in the intervention group will be facilitated by the use of family accelerometry data collected at baseline. It is intended that sharing of baseline physical activity data will encourage an awareness of the true rather than perceived levels of physical activity [38] and sedentary behaviour which may help motivate families to increase their physical activity. Sharing of baseline accelerometry data with the intervention group may also facilitate the setting and monitoring of more realistic physical activity and dog-walking goals by families. Families in the intervention group will also be shown a lay summary of baseline accelerometry data for their dog, using the method we have validated recently [39], to encourage a realistic understanding of current dog walking behaviour and baseline levels of physical activity of their dog.

The intervention will target parents and children being physically active together, using their pet dog to make this happen. It will consist of: promotion of child play with the dog by provision of a portfolio of suggested games as 'hide and seek' and 'obstacle course' to be played outdoors and indoors; provision of greater access to dog walking opportunities in the wider environment by providing intervention group families with information on dog walking routes, dog waste bins, and maps, describing outdoor spaces in the immediate environment and further afield; promotion of parental support for physical activity involving the dog, both modelling of physical activity and parental encouragement and logistical support; the provision of < £15 worth of physically active toys and dog walking equipment per family intended to facilitate play with the dog outdoors and more controlled walking with the dog, with guidance on the appropriate use of these materials from the Animal Behaviourist.

The intervention is intended to be generalisable, consisting of three home-based sessions (each approximately one hour, two in week 0 and one in week 6) with participating families and the researchers who will deliver the intervention, followed by four telephone contacts at weeks 2, 4, 8 and 10. Graded and individualised goals on dog walking and physical activity will be agreed and reviewed. One of the home-based sessions in week 0 with intervention families will be a 60-minute dog-walking/play/training session with the Certified Clinical Animal Behaviourist to help screen dogs and family for safety aspects of the intervention, provide support for the practical physical aspects of dog walking (e.g. best use of equipment) and to train families and dogs to interact in ways most likely to promote the physical activity of both family and dog. The intervention is outlined in brief in Table 2.

**Table 2:** Content of CPET Intervention

Week	Contact Type	Personnel	Content
0	Visit	Animal Behaviourist	Safety screening for family and dog; best practice of equipment use; practical training aspects of physical activity interactions with child and dog.

1	Visit	PARA	Overview of intervention timeline; decision balance; discussion of individual physical activities and sedentary behaviours; identifying alternative behaviours; goal setting; reward structure; self-monitoring (Activity Chart).

2	Telephone (verbal)	PARA	Review goal progress and self-monitoring; address other questions; review social support; provide positive reinforcement.

4	Telephone (text)	PARA	Statement of positive encouragement relating to individual goals plus a helpful hint to becoming more active.

6	Visit	PARA	Review goal progress, self-monitoring and rewards; relapse prevention.

8	Telephone (verbal)	PARA	Review goal progress, self-monitoring; address any questions; positive encouragement.

10	Telephone (text)	PARA	Statement of positive encouragement relating to individual goals plus reminder of forthcoming post- intervention measurements.

PARA: Physical Activity research assistant

The control group will receive the intervention after all of the outcome measures have been completed (a 'waiting list' control design).

### Outcome measures

Following recruitment, baseline measures will be made on all participants, and then participating families will be allocated randomly to intervention or control groups. Outcomes will be measured at baseline and 11 weeks later (in the week after the end of the intervention for the intervention group).

The UK Medical Research Council Framework for the development and evaluation of complex interventions in public health [33] recommends consideration of a potentially wide range and large number of outcomes at the exploratory trial stage which might be modified or reduced, in the light of exploratory trial experience, when the subsequent, more definitive, trial is planned. With this advice in mind the present study has included a large number of measures of a range of outcomes in the children, all of which are at least potentially sensitive to changes in physical activity [5]. The trial will also include measurement of a number of outcomes in parents and dogs. The primary and secondary outcomes in children, parents, and dogs are summarised in Table 3.

**Table 3:** Primary and secondary outcomes in children, parents and dogs

	Outcomes
**Children *Primary outcome***	• 10 week change (baseline-1 week post-intervention) in objectively measured total volume of physical activity with the Actigraph accelerometer (The Actigraph, Florida) using the accelerometry count per minute [40]; over 7 days *in the children*.

**Children *Secondary outcomes***	• Changes in objectively measured light intensity physical activity and MVPA (using the validated accelerometry cut-points of Puyau et al.; [41]);
	• Changes in objectively measured sedentary behaviour (using the validated Actigraph accelerometry cut-point of Puyau et al. [41]);
	• Changes in the patterning of sedentary behaviour (length of sedentary bouts, frequency of breaks in sedentary time; 7 day Actigraph accelerometer with the pragmatic cut-off, not yet validated and calibrated, of 150 counts per minute to define sitting time; 34);
	• Changes in body composition (fat mass index and lean mass index) based DXA using a Lunar Prodigy whole-body scanner (GE Medical Systems, Madison, WI) in conjunction with enCORE software version 13;
	• Changes in body weight and in BMI Z scores expressed relative to UK 1990 reference data; Changes in whole body and lumbar spine bone mineral content (DXA);
	• Changes in Child Health Related Quality of Life, as reported separately by both the children and by their parents, using the PedsQL which is practical, valid, and sensitive to change resulting from lifestyle interventions [37,42].

**Parents *Secondary outcomes***	• Changes in objectively measured physical activity (7 day Actigraph accelerometry) for total volume of physical activity, as well as light intensity physical activity and MVPA;
	• Changes in sedentary behaviour (total time and patterning of sitting time using the pragmatic cut-off of 100 Actigraph counts per minute to define sitting behaviour; 34);
	• Changes in parent body weight.

**Dog *Secondary outcomes***	• Changes in body condition score [43]; • Changes in total volume of physical activity (Actigraph accelerometry; 39), in part as a process evaluation measure.

### Process evaluation

In order to conduct a process evaluation of the intervention and trial, and to inform future interventions, we will evaluate the child-dog walking diary in combination with simultaneous Actigraph accelerometry data from parent, child, and dog (to identify whether and when all three were physically active together) at the end of the intervention. This will permit an assessment of the extent to which children, parents, and pets were 'exercising together' at baseline and at follow up, including a summary of the number and frequency of episodes when all three were physically active together in each week.

A brief study exit questionnaire with Likert scale responses will be used to obtain feedback on the intervention (as a form of process evaluation) from all families in the intervention group, to categorise those engaged versus those less engaged with the intervention (for the purposes of the per-protocol analysis), and to identify perceived barriers and facilitators of the intervention; perceptions of the acceptability of the intervention and the outcome measures and suggestions for future interventions.

A qualitative study, involving a focus group with participating parents and a separate focus group with participating children, will be conducted after the intervention in order to: inform the process evaluation of the intervention; obtain participant views on the acceptability of the intervention and outcome measures; obtain participant suggestions for the intervention to be developed for the larger, longer term, trial.

**Figure 1:**
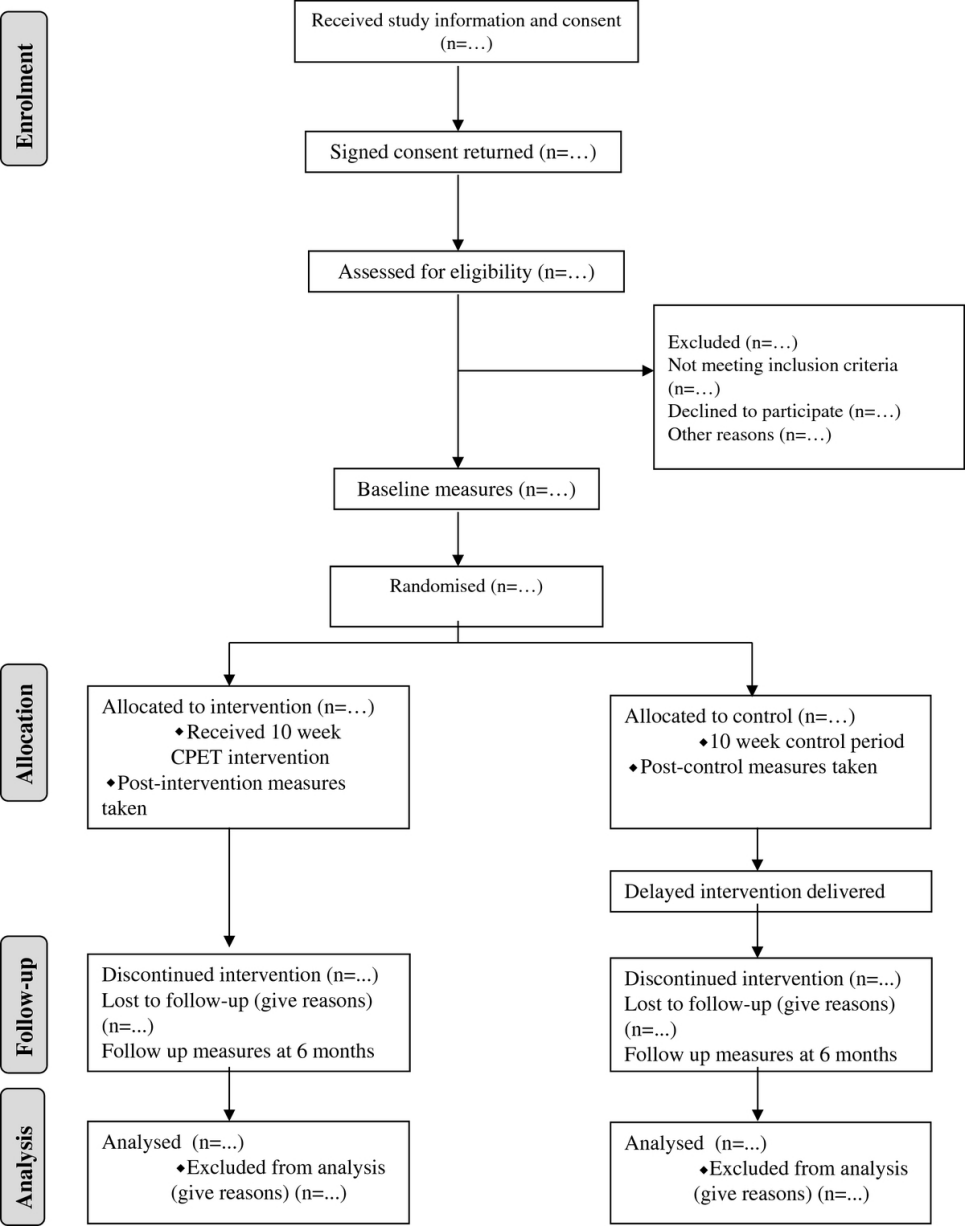
**Flow of study design showing potential attrition during enrolment, allocation, follow up and analysis**.

Finally, a CONSORT study flow diagram [36] (Figure 1) will be used to summarise sample attrition and missing data for all of the outcome measures.

## Discussion

### Study context

As noted above, there is considerable public health interest in the possibility of using pet dogs to promote health of humans in general, and to promote physical activity specifically. However, there is a dearth of intervention studies which aim to test whether and how pet dogs might be useful in physical activity promotion. Indeed, despite the great current interest in this topic as evidenced by many recent publications in the epidemiological and public health literature, no RCT of a public health intervention using pet dogs has yet been published. Physical activity is seen widely as one of the 'best bets' in public health strategy [44,45], yet relatively few well established interventions exist which will produce marked and sustained increases in physical activity in any population. There are few well founded family-based interventions despite the potential of modification of the family environment for promotion of physical activity in children [11,30-32]. Most physical activity promotion research to date with school-age children has been school-based, and has had mixed results [11,12]. Dog-based interventions can-potentially at least [18], address the problem of sustaining the intervention since the dog will remain with the family beyond the end of the intervention, and because subsequent dogs may join the family (and children may become dog owners as adults), possibly promoting a more physically active lifestyle which becomes long-term.

Using dogs to promote physical activity may also have wide public health 'reach'. In some western populations prevalence of pet dog ownership is as high as one-third of all households, and in sub-groups of these populations the prevalence of dog ownership is even higher. Pet dogs may be useful in promoting physical activity of all members of the family: children; adolescents; adults. Dog ownership is so common that most children in western societies have at least potential access to dogs via extended families, friends, or neighbours. Dog-based interventions may also be helpful in special populations, in secondary prevention for example [29].

Examining whether the potential of pet dogs can be realised in public health will require the rigorous testing of interventions using RCT which have been developed according to helpful logic frameworks such as that provided by the UK Medical Research Council and these in turn will depend on the exploratory trials of the kind described here.

### Study strengths and weaknesses

The present study has a number of strengths. It takes a logical approach to the development and evaluation of the intervention, based on the UK Medical Research Council Framework, the intervention itself is theory based, and the study is aimed at providing high level evidence. It targets the common and important public health problems of low levels of physical activity, high levels of sedentary behaviour and high prevalence of obesity, with a logical emphasis on a stage of the lifecycle (mid-late childhood) recently demonstrated to be a public health problem in the UK [4,7,9,10]. Dissemination of the study and the intervention are priorities for the research team, in order to facilitate future interventions of this kind, as evidenced by the current and subsequent research reports, so the intervention materials will also be available subsequently from the corresponding author. The trial has been registered, in part to facilitate dissemination. The study also has access to a large number of state of the art outcome measures of a range of variables. The study is multi-disciplinary, involving collaborators from an unusually wide range of specialties, with expertise in childhood measurement methodology and trial design and conduct, behavioural science as applied to physical activity interventions with adults and children, human-dog interactions in public health, dog behaviour, veterinary medicine and dog physical activity, and biostatistics. Exceptionally in this field of research the present study is designed to consider equally both the people and the pets involved, including the behavioural welfare of the animals being used in the public health intervention. This collaboration is unusually broad in scope but essential in order to design and develop the intervention, to evaluate it, to progress it along the path outlined by the UK Medical Research Council Framework and to disseminate it effectively. Finally, the trial will include a process evaluation and a qualitative study in order to inform the development of the future intervention to be tested in a future more definitive trial, as recommended by the UK Medical Research Framework [33].

The present study has a number of weaknesses. It lacks an economic evaluation, though this is arguably more appropriate as part of a more definitive trial in future. The present study is intended to test a relatively simple and potentially generalisable intervention. It is possible that an intervention with a higher 'dose' of input might have greater efficacy, but possibly at the expense of reduced generalisability. While extensive expert input has gone into the design of the present study intervention, and the intervention itself uses a resource not available widely (a home visit by a Certified Clinical Animal Behaviourist), in the longer term the trial is intended to facilitate future interventions which are simpler and more accessible, by dissemination of trial lessons and materials more widely via the internet, and/or a book or DVD. The challenges and barriers observed during this pilot will be crucial to informing the design of future interventions. Longer-term follow up to test the sustainability of any intervention effects would have been desirable, but the short-term follow up in the present study is probably adequate for an exploratory trial: In a future definitive trial longer-term follow up should be included. The 'reach' of dog-based health promotion interventions may be seen by some as limited, but in fact they probably have a higher reach than many public health interventions to date (with the exception of school-based interventions), and might in future be considered instead of, or in addition to, other interventions. Experience with CPET might also inform family based physical activity promotion more generally, an approach which is promising but which has a limited evidence base to date [11]. The future reach of dog based interventions into socioeconomically deprived groups in the UK is potentially high given the disproportionately high prevalence of dog ownership in more deprived families [19,46]. While the primary outcome of the present study is physical activity, dog-based interventions might in fact have a greater impact on other outcomes of considerable public health importance, such as total time spent sedentary, or the patterning of sedentary behaviour [34,47-50]. These relatively new constructs have been included in the list of outcome measures in the present study and, depending on the results, might assume greater importance in the more definitive trials which should follow.

## Conclusions

The present study will be the first family-based RCT aimed at promotion of physical activity in children using pet dogs as the basis of the intervention. The study should form a helpful basis for future public health interventions which aim to use dogs to promote physical activity and/or reduce sedentary behaviour in children, adolescents, and adults. It may also be helpful in the design of future dog-based interventions aimed at special populations in secondary prevention or in treatment: in the treatment of child or adult obesity for example [29,37] where adherence to the physical activity prescription is generally poor [37].

## Abbreviations

ALSPAC: Avon Longitudinal Study of Parents and Children; BMI: Body mass index; CPET: Children parents and Pets Exercising Together; DXA: Dual energy X-ray absorptiometry; RCT: Randomised controlled trial.

## Competing interests

The authors declare that they have no competing interests.

## Authors' contributions

VP and RM have determined outcome measures, study protocol and had input into the paper. PH and CW have advised re canine behaviour and suggested inclusion criteria and developed a suitable questionnaire. NM and DW have contributed to study design. JR and PY conceived the study, and participated in its design and coordination. All authors were involved in study design and methods. All authors were involved in drafting and redrafting the manuscript. All authors read and approved the final manuscript.

## Pre-publication history

The pre-publication history for this paper can be accessed here:

http://www.biomedcentral.com/1471-2458/12/208/prepub
